# Characterization of the complete chloroplast genome of *Ginkgo biloba* L. (Ginkgoaceae), an endangered species endemic to China

**DOI:** 10.1080/23802359.2019.1692720

**Published:** 2019-11-20

**Authors:** Peng Jiao, Zhuo Qi, Zhenzhong Jiang, Jing Qu, Shuyan Guan

**Affiliations:** College of Life Sciences, Jilin Agricultural University, Changchun, Jilin, China

**Keywords:** *Ginkgo biloba* L., endangered species, chloroplast genome

## Abstract

*Ginkgo biloba* L. is the oldest relict plant among the gymnosperms, left after the quaternary glacial movement. There are few living *G. biloba* and few old trees over a hundred years old. It is currently on the International Union for Conservation of Nature (IUCN) red list of threatened species. In this study, we first assembled the complete chloroplast (cp) genome of *G. biloba* L. by Illumina paired-end reads data. The whole genome was 156,988 bp, consisting of a pair of inverted repeats of 34,056 bp, large and small single copy regions of 56,819 and 22,763 bp in length, respectively. The cp genome contained 131 genes, including 84 protein-coding genes, 31 trRNA genes and 5 rRNA genes. The overall GC content of the whole genome was 39.6%. A neighbour-joining phylogenetic analysis demonstrated a close relationship between *G. biloba* L. and *Cycas revoluta*.

Ginkgo is a very characteristic tree species, with edible seeds, extremely strong medicinal efficacy and ornamental value (Pang et al. 1996). *Ginkgo biloba* is the only surviving species in the ginkgo family, and most of its natural habitats are located in China (Shen et al. 2005). The Chinese endemic species is also a national I level key protected wild plants, in addition to Heilongjiang, Inner Mongolia, Qinghai, Tibet, the provinces, area all have distribution, in the majority with Jiangsu, Shandong, Zhejiang, and Guangxi. This plant species is under serious threat by human activities and overexploitation for ornamental value and economic uses (Pei 2015). Therefore, scientists call them “living fossils” and “pandas of the plant world” (Farjon 2013). At present, the number of *Ginkgo* trees is decreasing, mainly due to deforestation.

The present study is the first time to assemble and characterize the complete chloroplast genome for *Ginkgo biloba* L. (GenBank: NC_016986.1) from high throughput sequencing data. The existing chloroplast Genome sequence of *G. biloba* was downloaded from the National Center for Biotechnology Information’s Organelle Genome Resources database (nc_016986.1) as the reference sequence, and the chloroplast Genome of *G. biloba* was assembled using SPAdes v3.6.0 software (Bankevich et al. 2012). The default setting of parameters was adopted. Sequence annotation first confirmed the availability and boundary of genes by BlastN comparison directly through the protein-coding sequence of the proximal species. Then, the genes in the chloroplast genome were annotated by online tool DOGMA (http://dogma.ccbb.utexas.edu/) with default parameters, and the genes were functionally annotated by combining with NR (http://www.ncbi.nlm.nih.gov/) database (Lohse et al. 2013). TRNA was annotated using the trnascan-se online site. Using RNAmmer 1.2 Server (HTTP//www.cbs.dtu.dk/services/RNAmmer/) rRNA for comments (Katoh and Standley 2013). The chloroplast genome of *G. biloba* was mapped using OGDRAW (HTTP//OGDRAW. Mpimp-golm. mpg. DE/cgi-bin/OGDRAW. Pl) software (Asaf et al. 2017).

The complete cp-DNA of *Ginkgo biloba* L. was a circular molecule 156,988 bp in length, comprising a large single copy (LSC) region of 56,819 bp and a small single copy(SSC) region of 22,763 bp, separated by two inverted repeat regions (IRs) of 34,056 bp (Figure 1). It contained 131 genes, including 84 protein-coding genes, 5 ribosomal RNA genes, and 31 tRNA genes. The phylogenetic tree reveals all the species of Pinaceae formed a monophyletic clade with high-resolution value and *Cycas revoluta* is highly related with *Ginkgo biloba* L. (Figure 1).

**Figure 1.**
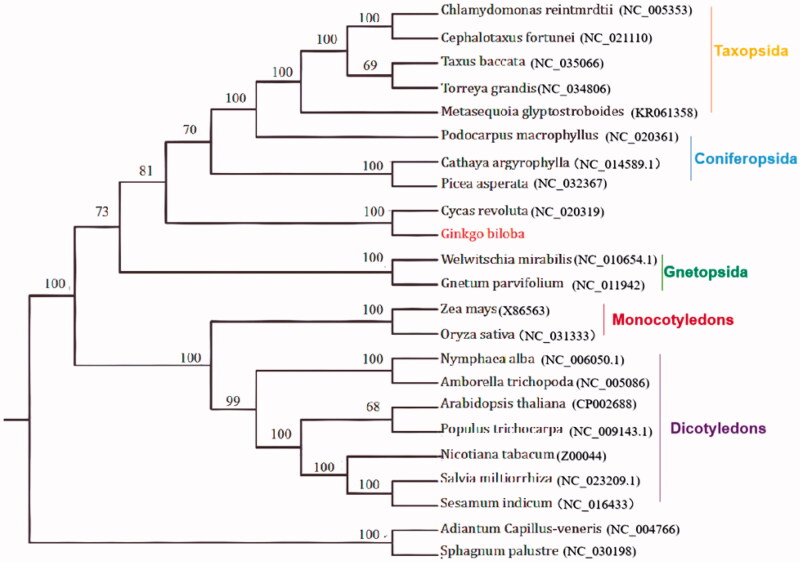
The phylogenetic tree based on 23 complete plastid genome sequences.
